# Supplementary material for: Systematic screening of generic drugs for progressive multiple sclerosis identifies clomipramine as a promising therapeutic
Source: Nat Commun. 2017 Dec 19;8:1990. doi: 10.1038/s41467-017-02119-6 (PMC5736601; doi:10.1038/s41467-017-02119-6)
Supplement: Supplementary file 1 — Supplementary Information [file 41467_2017_2119_MOESM1_ESM.pdf]

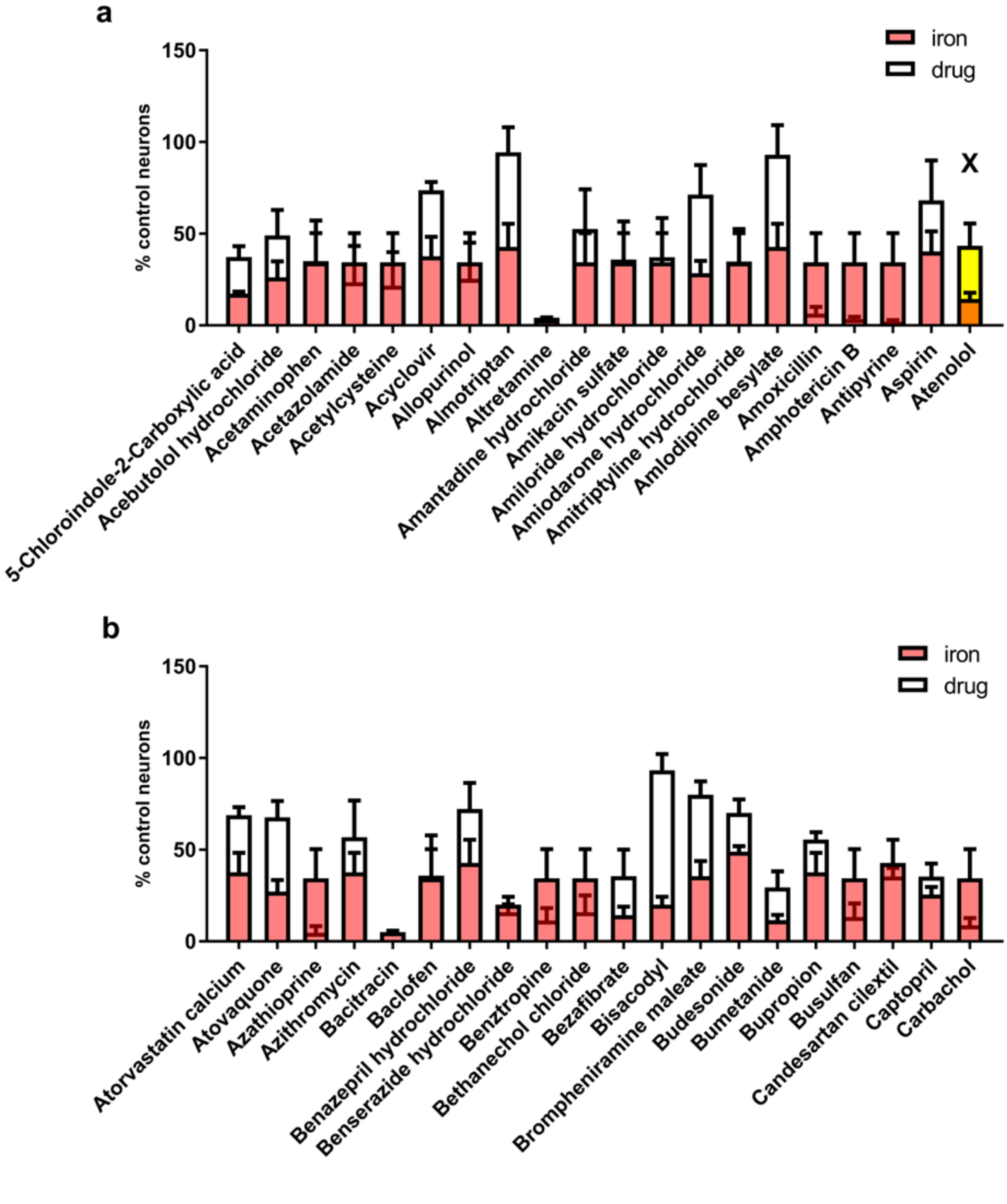

Supplementary Figure 1 – Summary of the screening of compounds against iron mediated neurotoxicity. The number of neurons left following exposure to each compound was normalized to the number of neurons of the respective control condition. The corresponding iron situation was also normalized to the respective control (red). Compounds which exhibit significant protection are highlighted in yellow and marked (X). Shown are the means ± SEM of 1-4 experiments, each performed in quadruplicates. Supplementary figures 1-7 comprise the complete list of medications tested for the iron-mediated neurotoxicity screening.

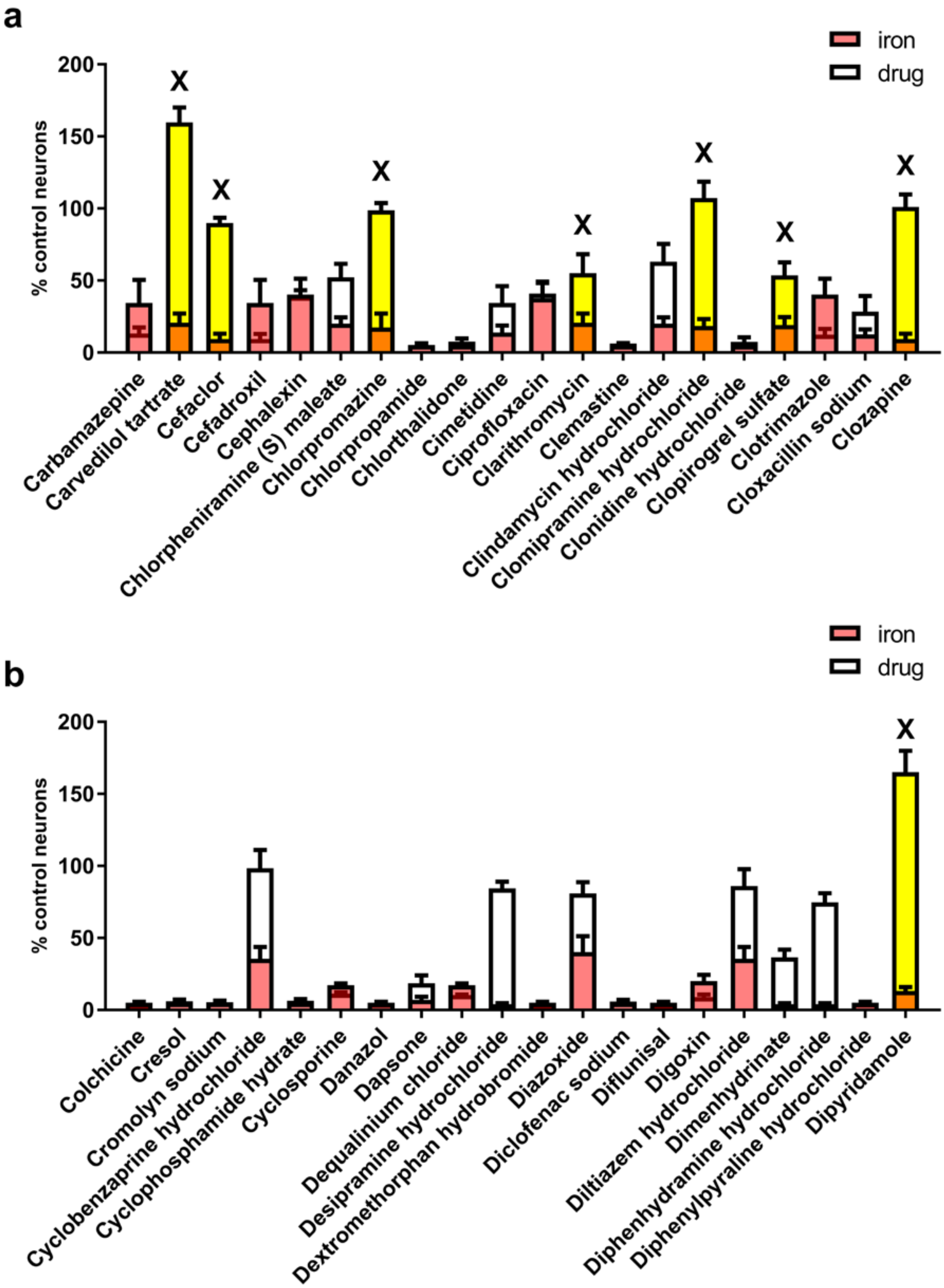

Supplementary Figure 2 – Summary of the screening of compounds against iron mediated neurotoxicity. The number of neurons left following exposure to each compound was normalized to the number of neurons of the respective control condition. The corresponding iron situation was also normalized to the respective control (red). Compounds which exhibit significant protection are highlighted in yellow and marked (X). Shown are the means ± SEM of 1-4 experiments, each performed in quadruplicates. Supplementary figures 1-7 comprise the complete list of medications tested for the iron-mediated neurotoxicity screening.

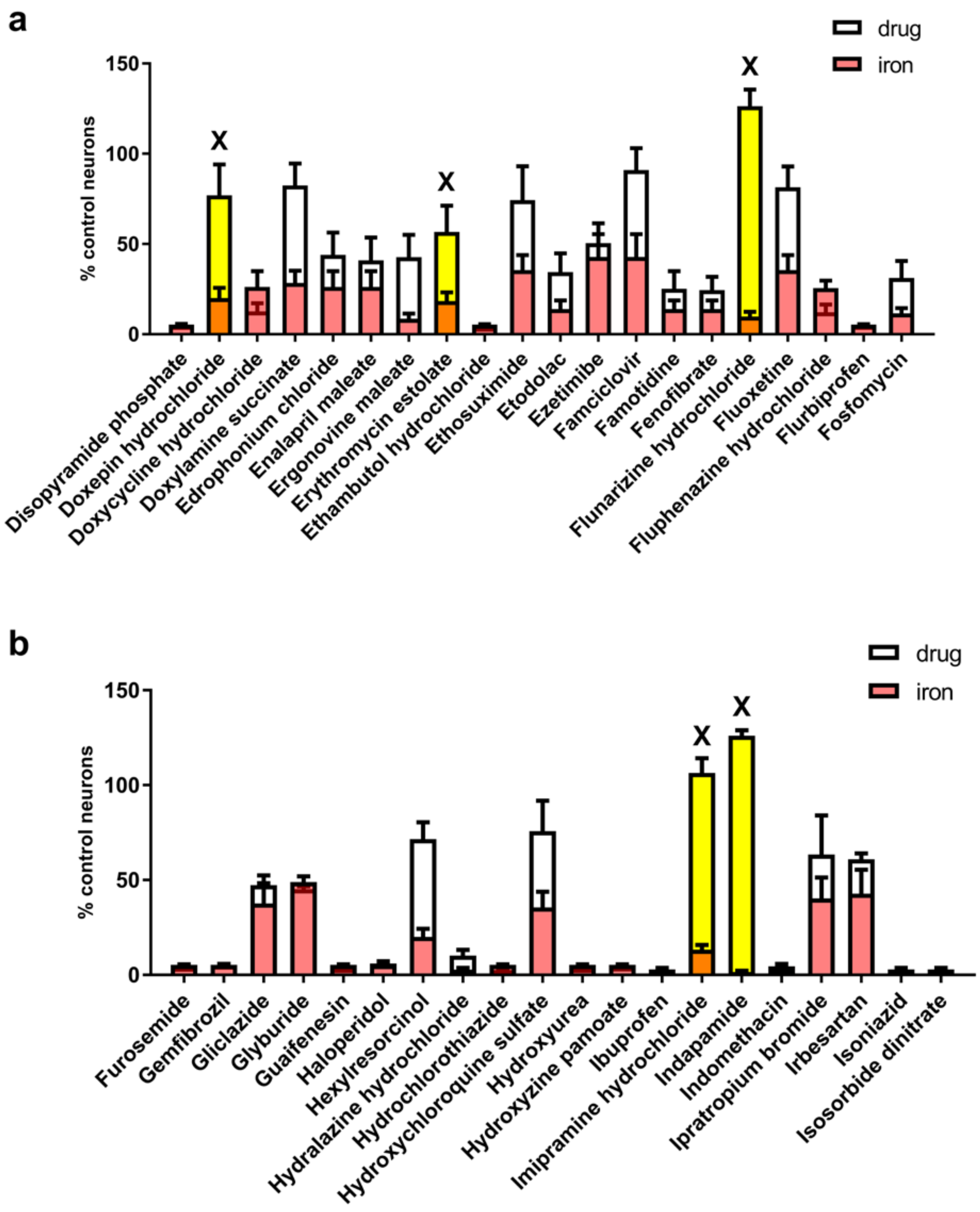

Supplementary Figure 3 – Summary of the screening of compounds against iron mediated neurotoxicity. The number of neurons left following exposure to each compound was normalized to the number of neurons of the respective control condition. The corresponding iron situation was also normalized to the respective control (red). Compounds which exhibit significant protection are highlighted in yellow and marked (X). Shown are the means ± SEM of 1-4 experiments, each performed in quadruplicates. Supplementary figures 1-7 comprise the complete list of medications tested for the iron-mediated neurotoxicity screening.

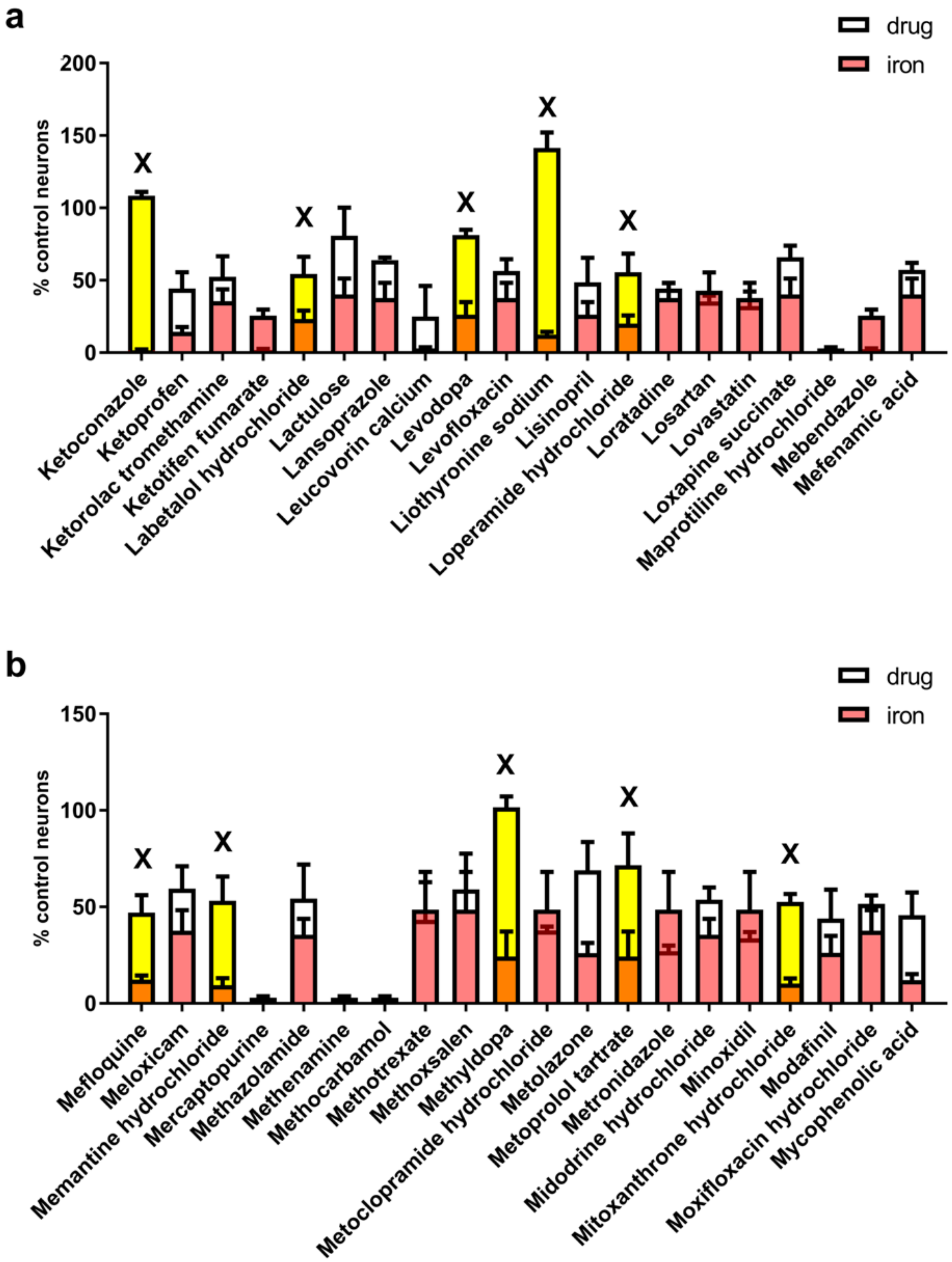

Supplementary Figure 4 – Summary of the screening of compounds against iron mediated neurotoxicity. The number of neurons left following exposure to each compound was normalized to the number of neurons of the respective control condition. The corresponding iron situation was also normalized to the respective control (red). Compounds which exhibit significant protection are highlighted in yellow and marked (X). Shown are the means ± SEM of 1-4 experiments, each performed in quadruplicates. Supplementary figures 1-7 comprise the complete list of medications tested for the iron-mediated neurotoxicity screening.

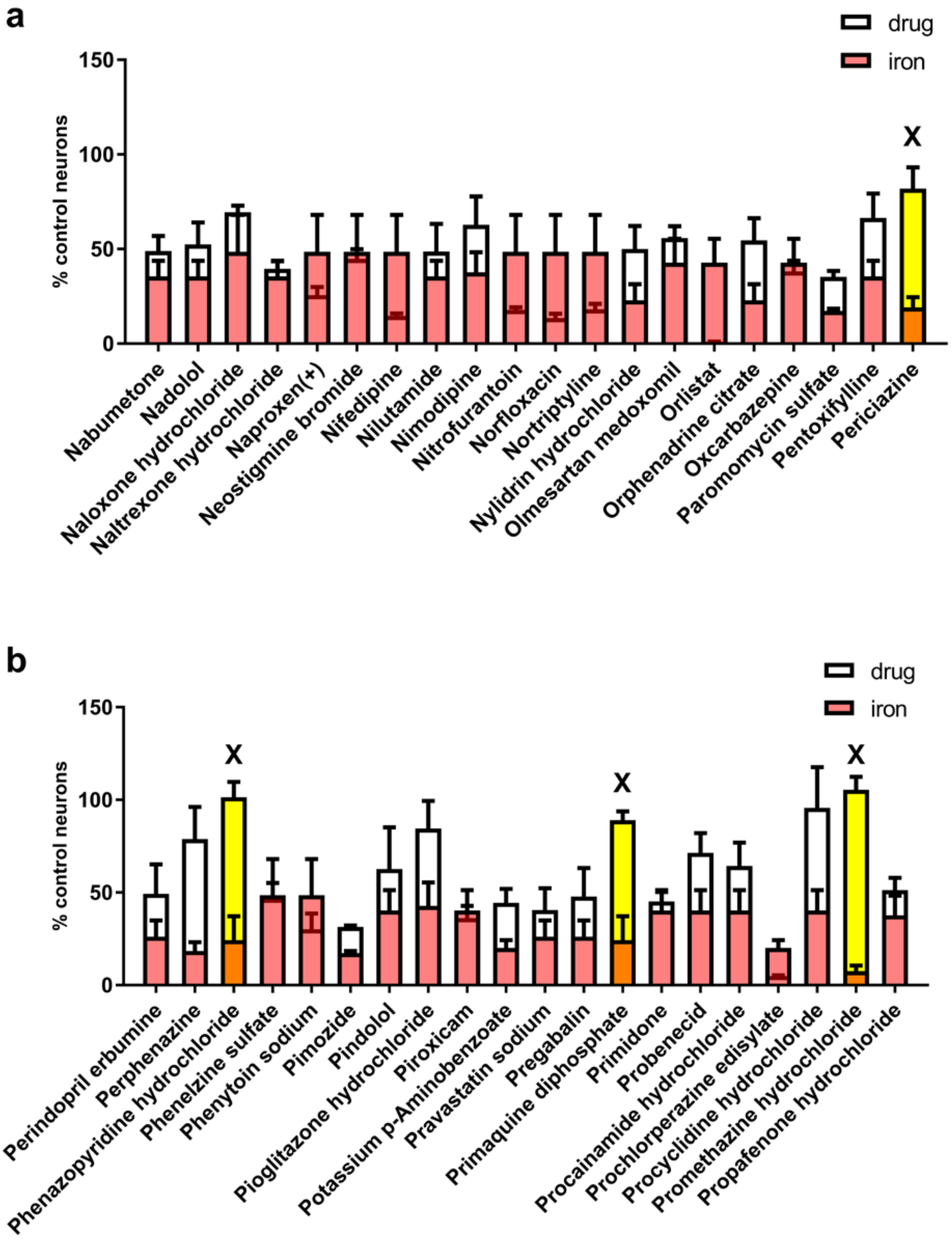

Supplementary Figure 5 – Summary of the screening of compounds against iron mediated neurotoxicity. The number of neurons left following exposure to each compound was normalized to the number of neurons of the respective control condition. The corresponding iron situation was also normalized to the respective control (red). Compounds which exhibit significant protection are highlighted in yellow and marked (X). Shown are the means ± SEM of 1-4 experiments, each performed in quadruplicates. Supplementary figures 1-7 comprise the complete list of medications tested for the iron-mediated neurotoxicity screening.

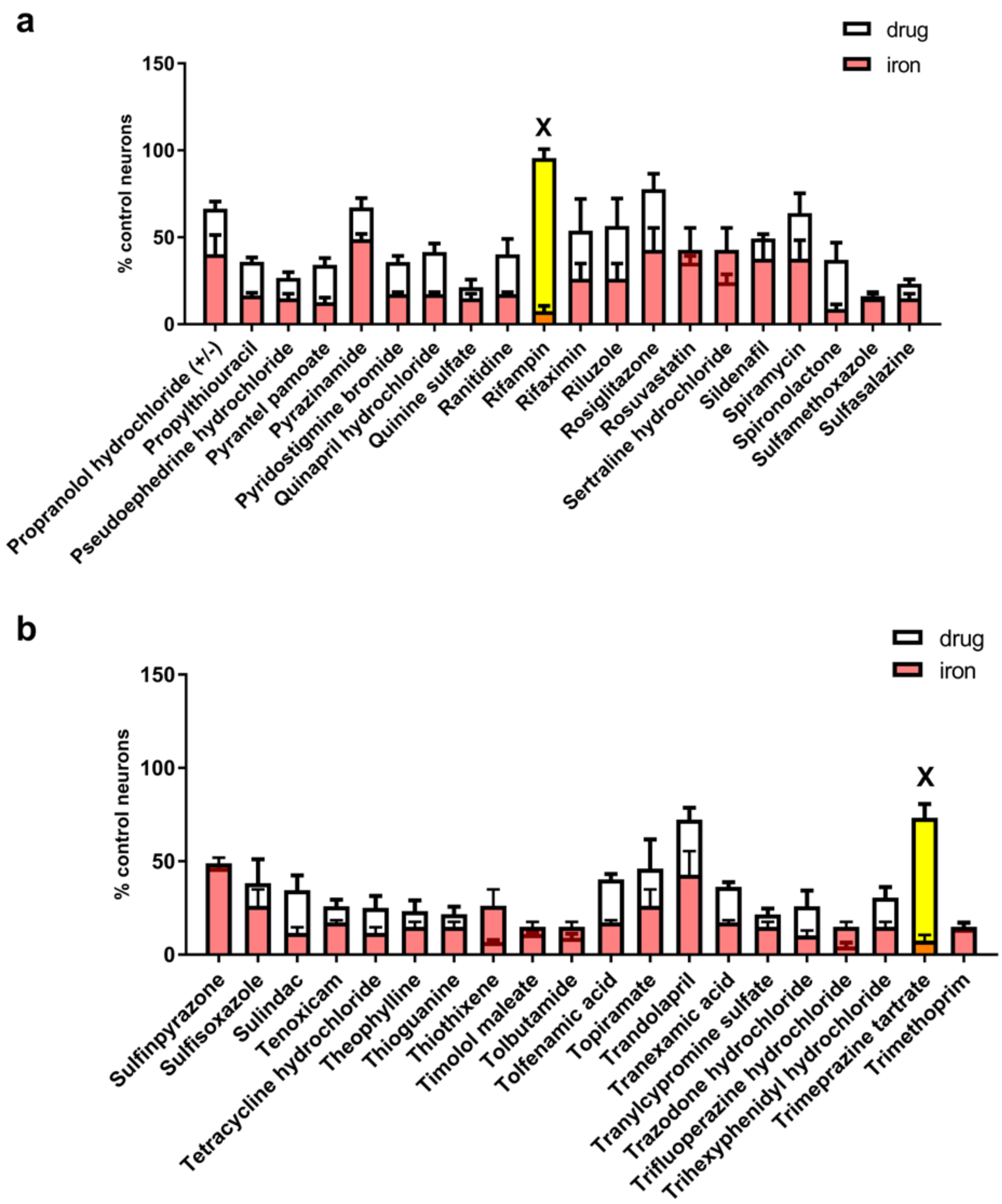

Supplementary Figure 6 – Summary of the screening of compounds against iron mediated neurotoxicity. The number of neurons left following exposure to each compound was normalized to the number of neurons of the respective control condition. The corresponding iron situation was also normalized to the respective control (red). Compounds which exhibit significant protection are highlighted in yellow and marked (X). Shown are the means ± SEM of 1-4 experiments, each performed in quadruplicates. Supplementary figures 1-7 comprise the complete list of medications tested for the iron-mediated neurotoxicity screening.

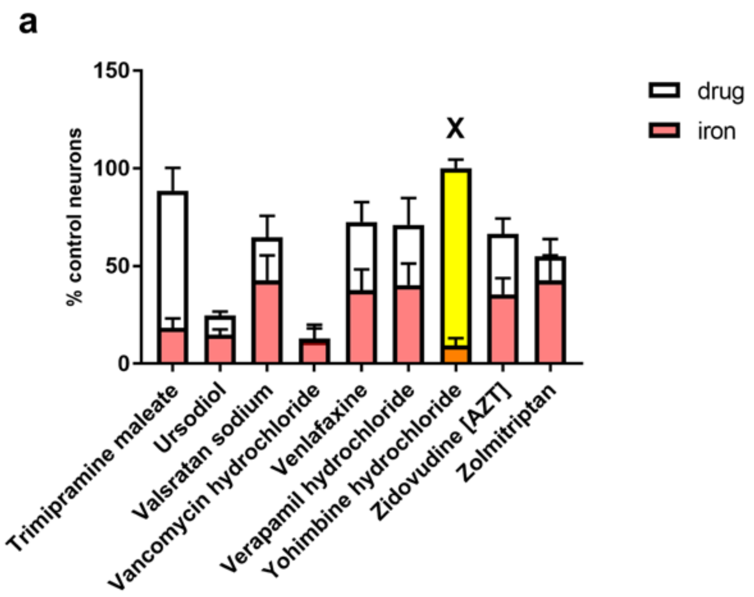

Supplementary Figure 7 – Summary of the screening of compounds against iron mediated neurotoxicity. The number of neurons left following exposure to each compound was normalized to the number of neurons of the respective control condition. The corresponding iron situation was also normalized to the respective control (red). Compounds which exhibit significant protection are highlighted in yellow and marked (X). Shown are the means ± SEM of 1-4 experiments, each performed in quadruplicates. Supplementary figures 1-7 comprise the complete list of medications tested for the iron-mediated neurotoxicity screening.
