# Supplementary material for: Systematic screening of generic drugs for progressive multiple sclerosis identifies clomipramine as a promising therapeutic
Source: Nat Commun. 2017 Dec 19;8:1990. doi: 10.1038/s41467-017-02119-6 (PMC5736601; doi:10.1038/s41467-017-02119-6)
Supplement: Supplementary file 3 — Description of Additional Supplementary Files [file 41467_2017_2119_MOESM3_ESM.pdf]

Description of Additional Supplementary Files

File Name: Supplementary Movie 1
Description: Live cell imaging of selected compounds that prevent iron mediated neurotoxicity. Shown is the live cell imaging of neurons, challenged with $FeSO_4$ in a concentration of 50 µM (b). Upon pre-treatment with indapamide (c) or desipramine (d) 1 h before the addition of iron, the number of propidium-iodide positive cells was significantly reduced after 7.5 h and even below the level of the control condition after 12 h, suggesting a strong neuroprotective effect (see Fig. 2b for analysis). Live cell imaging was performed over 12 h; images were taken every 30 min. Neurons were stained with Hoechst 33342 (cyan) and propidium-iodide (red). For better presentation, videos were edited using the Image J software.

File Name: Supplementary Movie 2
Description: Live cell imaging of clomipramine against iron-mediated neurotoxicity. Shown is the live cell imaging of neurons, challenged with $FeSO_4$ in a concentration of 50 µM (b). Upon pre-treatment with 2 µM clomipramine (c) 1 h before the addition of iron, the number of propidium-iodide positive cells was significantly reduced after 5 h, suggesting a strong neuroprotective effect (see Fig. 6c for analysis). Panel a) shows the untreated control. Live cell imaging was performed over 6.5 h, images were taken every 30 min. Neurons were stained with Hoechst 33342 (cyan) and propidium-iodide (red). For better presentation, videos were edited using the Image J software.

File Name: Supplementary Data 1
Description: Shown is a summary of the 249 compounds analyzed with ID, name, plate, position (as named by manufacturer), formula, molecular weight, bioactivity and source, as provided by MicroSource Discovery. The designation of each drug on the plates was previously described 14. INN: International Nonproprietary Name; JAN: Japanese Accepted Name; USAN: United States Adopted Names; USP: United States Pharmacopeia.

File Name: Supplementary Data 2
Description: Shown are the results of the iron-mediated neurotoxicity screen of all 249 compounds. The number of neurons of the respective drug treatment and the corresponding iron situation were normalized to the number of neurons of the control condition and presented as mean ± SEM. N=1 to 4 independent experiments, each performed in quadruplicates.
